# Transplantation of human induced pluripotent stem cell-derived cardiomyocytes improves myocardial function and reverses ventricular remodeling in infarcted rat hearts

**DOI:** 10.1186/s13287-020-01602-0

**Published:** 2020-02-21

**Authors:** Xumin Guan, Wanzi Xu, He Zhang, Qian Wang, Jiuyang Yu, Ruyi Zhang, Yamin Chen, Yunlong Xia, Jiaxian Wang, Dongjin Wang

**Affiliations:** 1 Department of Cardiology, The First Affiliated Hospital of Dalian Medical University, Dalian, 116011 Liaoning China; 2 Department of Thoracic and Cardiovascular Surgery, Nanjing Drum Tower Hospital, Clinical College of Traditional Chinese and Western Medicine, Nanjing University of Chinese Medicine, Nanjing, 210008 Jiangsu China; 3 Department of Thoracic and Cardiovascular Surgery, Peking Union Medical College Nanjing Drum Tower Hospital, Nanjing, 210008 Jiangsu China; 4 HELP Therapeutics, Nanjing, 211166 Jiangsu China; 5 The Laboratory Animal Center, The First Affiliated Hospital of Nanjing Medical University, Nanjing, 210029 Jiangsu China; 6 Department of Cardiology, The First Affiliated Hospital of Nanjing Medical University, Nanjing, 210029 Jiangsu China; 7 Department of Cardio-Thoracic Surgery, Nanjing Drum Tower Hospital Affiliated to Medical School of Nanjing University, Nanjing, China

**Keywords:** Human induced pluripotent stem cell, Cardiomyocytes, Regenerative medicine, Remodeling, Heart failure

## Abstract

**Background:**

Human-induced pluripotent stem cell-derived cardiomyocytes (iPSC-CMs) have shed great light on cardiac regenerative medicine and specifically myocardial repair in heart failure patients. However, the treatment efficacy and the survival of iPSC-CMs in vivo after transplantation have yielded inconsistent results.

**Objectives:**

The objective of this study was to evaluate the ability of human iPSC-CMs to improve myocardial function in a rat postinfarction heart failure model.

**Methods:**

Eight-week-old male Sprague-Dawley rats were randomly selected to receive an intramyocardial injection of 5% albumin solution with or without 1 × 10^7^ human iPSC-CMs 10 days after undergoing left anterior descending (LAD) coronary artery ligation. Cyclosporine A and methylprednisolone were administered before iPSC-CM injection and until the rats were killed to prevent graft rejection. Cardiac function was evaluated by echocardiography. The survival of grafted cardiomyocytes was confirmed by observing the fluorescent cell tracer Vybrant™ CM-DiI or expression of the enhanced green fluorescent protein (eGFP) in transplanted cells, or survival was demonstrated by polymerase chain reaction (PCR)-based detection of human mitochondrial DNA. Sirius red stain was used to evaluate the fibrosis ratio. Hematoxylin-eosin staining was used to observe the formation of teratomas.

**Results:**

Four weeks after intramyocardial injection of iPSC-CMs, animals undergoing iPSC-CM transplantation had lower mortality than the control group. Animals injected with cell-free solution (control group) demonstrated significant left ventricular (LV) functional deterioration, whereas grafting of iPSC-CMs attenuated this remodeling process. In the control group, the ejection fraction deteriorated by 10.11% (from 46.36 to 41.67%), and fractional shortening deteriorated by 9.23% (from 24.37 to 22.12%) by 4 weeks. In the iPSC-CM injection group, the ejection fraction improved by 18.86% (from 44.09 to 52.41%), and fractional shortening improved by 23.69% (from 23.08 to 28.54%). Cell labeling, tracking, and molecular biology techniques indicated that the grafted cardiomyocytes survived in the rat heart 1 month after iPSC-CM transplantation. Myocardial fibrosis was also attenuated in the iPSC-CM treatment group.

**Conclusions:**

Human iPSC-CM grafts survived in infarcted rat hearts and restored myocardial function 4 weeks after transplantation. Cell replacement therapy also reversed ventricular remodeling, indicating the potential of iPSC-CMs for cardiac repair strategies.

**Electronic supplementary material:**

The online version of this article (10.1186/s13287-020-01602-0) contains supplementary material, which is available to authorized users.

## Background

Heart failure remains the leading cause of morbidity and mortality worldwide [1]. In patients with chronic heart failure, especially in those with III-IV symptoms, as defined by the New York Heart Association, the prognosis is extremely poor due to irreversibly impaired left ventricular function [2]. The treatment of heart failure remains a challenging problem as conventional treatments (drug therapy, interventional therapy, and surgery) have difficulty in restoring the function of the heart. Myocardial infarction (MI), the most common heart disease, is characterized by a significant reduction in the number of functional cardiomyocytes and leads to the development of progressive heart failure. Adult hearts have little ability to regenerate [3] and traditional treatments fail to address the fundamental problem of muscle deficiency. Heart regenerative therapy, such as transplantation of myocytes [4] and cardiomyocytes to replace lost cells, has become a new strategy for the treatment of heart failure caused by MI [5].

Pluripotent stem cells (PSCs), including embryonic stem cells (ESCs) and induced pluripotent stem cells (iPSCs), are potential sources of therapeutic cardiomyocytes [6–8]. They have the capacity for unlimited proliferation in vitro while maintaining the potential to differentiate into derivatives of the three germ layers. A well-established method of cardiomyocyte differentiation from PSCs provides an ideal source of cardiomyocytes and makes regenerative therapy for myocardial muscle damage a promising strategy. The methods of cardiomyocyte differentiation, purification, and maturation from iPSCs or ESCs have been established both by our lab [9–12] and other labs [13–17]. Recently, transplantation of human ESC-derived cardiomyocytes (ESC-CMs) has been proven to significantly improve cardiac function in infarcted rat and nonhuman primate hearts [18, 19] due to the capacity of PSC-CMs to remuscularize the infarcted hearts and form electromechanical junctions with the host hearts [15]. Human iPSC-CMs have great potential in disease treatment because they avoid the social ethical issues of ESCs and avoid the possibility of immune rejection [20]. Although some investigators reported that transplantation of iPSC-CMs reduced remodeling of the heart after ischemic damage [21–24], the field is still at the preclinical stage due to technical and surgical hurdles. In addition, the treatment efficacy and the survival of iPSC-CMs in vivo after transplantation is debatable. Chow et al. demonstrated that no grafted iPSC-CMs were detected after 1 month of transplantation [22]. In the present study, we aimed to evaluate the ability of human iPSC-CM transplantation to improve myocardial function in the rat MI model and to determine the fate of transplanted cells in the rat heart.

## Methods

### Differentiation of iPSC-CMs and cell preparation

Human iPSC line was derived from a healthy man (32 years old) and transduced with Oct3/4–Sox2, cMyc, and Klf4. Informed consent was obtained from the donor prior to all experiments. The iPSC reprogramming and cardiomyocytes manufacturing were conducted under GMP-grade lab at HELP Therapeutics. Undifferentiated human iPSCs were grown to 90% confluence and subsequently differentiated into beating cardiomyocytes. In brief, on day 0 and day 1, iPSCs were given #1 medium (RPMI 1640 [Gibco] and B27 supplement minus insulin [Gibco]) supplemented with 6 μM CHIR-99021 [Sigma-Aldrich], which is a selective inhibitor of glycogen synthase kinase 3β that activates the canonical Wnt signaling pathway. On day 2, the medium was replaced with #1 medium without CHIR99021. On day 3 and day 4, cells were supplemented with #1 medium with 5 μM of IWR-1 [Sigma-Aldrich], which is a Wnt antagonist. Then, the medium was replaced with #1 medium every day until day 8, and the medium was replaced with #2 medium (RPMI 1640 [Gibco] and B27 supplement [Gibco]) every other day. Usually, the cells began to have spontaneous contractions after 8–10 days of differentiation. On days 16–20 of differentiation, purified iPSC-CMs were dissociated and 1 × 10^7^ cells were cryopreserved per cryogenic tube. Cryopreserved cardiomyocytes were thawed in a 37 °C water bath (for approximately 2 min) and suspended in 75 μl 5% albumin solution before transplantation.

### Animal model of MI and cell transplantation

The experimental flow chart is shown in supplementary Figure 1. After undergoing a left thoracotomy, 8-week-old male Sprague-Dawley rats (approximately 250 g) underwent MI by ligation of the left anterior descending (LAD) coronary artery 10 days before receiving an injection of cells. Electrocardiogram (ECG) data were used to confirm the establishment of the MI model by ST segment elevation (Fig. 2c, d). Ten days after MI, the rats underwent echocardiography, and a second thoracotomy was performed in the rats that met the inclusion criteria: Ejection fraction was reduced by between 15 and 50%. Then, 1 × 10^7^ human iPSC-CMs in 5% albumin solution or a control solution containing only 5% albumin were transplanted to the infarcted area and infarcted margins at two to three different sites. Two groups were studied: (1) a control group in which 5% albumin solution (75 μl) was injected (*n* = 13) and (2) a group in which 1.0 × 10^7^ iPSC-CMs in 5% albumin solution were grafted (*n* = 18). To prevent graft rejection, animals from all groups were treated with cyclosporine A (15 mg/kg/day) and methylprednisolone (2 mg/kg/day) from the day before iPSC-CM delivery until rats were dissected. Most rat hearts were harvested 4 weeks later (day 28).

### Echocardiography

As shown in supplementary Figure 1, transthoracic echocardiography was performed at baseline (before MI model established), day 0 (10 days after LAD coronary artery ligation and before iPSC-CM grafting), and day 28 (4 weeks after iPSC-CM grafting) using the Vevo LAB 3.1.0 (FUJIFILM VisualSonics, Inc.). The following parameters were measured: (1) left ventricular end-diastolic and end-systolic diameters (LVEDD and LVESD, respectively); (2) left ventricular end-diastolic and end-systolic volume (LVEDV and LVESV, respectively); (3) ejection fraction (EF); (4) fractional shortening (FS); (5) left ventricular end-diastolic and end-systolic anterior wall thickness. All measurements were averaged over three cardiac cycles and assessed by an experienced operator blinded to the treatment group.

### Tracking of iPSC-CMs

To identify the transplanted cells, we used a number of labeling, tracking, and molecular biological techniques: (1) labeling with the fluorescent cell tracer Vybrant™ CM-DiI (5 μM, Molecular Probes, Invitrogen, V22888); (2) tagging with the genetic marker enhanced green fluorescent protein (eGFP), the introduction of which was achieved using lentiviral transduction; (3) measuring human mitochondrial DNA via polymerase chain reaction (PCR) and qPCR at different times following transplantation (supplementary Figure 1).

### Histological examination

For histological examination, hearts were fixed with 4% paraformaldehyde in PBS for Sirius red staining or HE staining and cryosectioned for immunostaining. For immunostaining, fixed hearts were immersed in 30% sucrose overnight, embedded in an optimal cutting temperature compound, frozen, and cryosectioned (4.5-μM sections). Immunostaining was performed, and primary antibodies were used at dilutions of 1:200 for anti-sarcomeric alpha-actinin (Abcam, ab137346) and 1:100 for anti-GFP (Abcam, ab1218). Secondary antibodies, including goat anti-rabbit IgG (H + L) (Alexa Fluor Plus 555, Invitrogen, A32732), were used at dilutions of 1:500 for detecting alpha-actinin, and goat anti-mouse IgG (H + L) (Thermo Fisher, F2761) was used at dilutions of 1:500 for detecting eGFP. Slides were imaged with an inverted fluorescence microscope (Leica inverted microscope DMi8).

### PCR and qPCR analysis

The rats were dissected at 24 h, 7 days, and 28 days after cell grafting. For PCR and qPCR, hearts and other organs were frozen in liquid nitrogen. The presence of iPSC-CMs within the rat hearts or other organs was evaluated using PCR and qPCR-based amplification of human mitochondria DNA. Genomic DNA was produced using the DNeasy Blood & Tissue Kit (Qiagen, 69504). PCR was carried out using Platinum™ Green Hot Start PCR Master Mix (Invitrogen™, 13001012). The sequence of the forward primer was CACCGGCGCAGTCATTCTCATA, and the reverse primer sequence was GAGTCCTGTAAGTAGGAGA.

### Statistical analysis

Data are expressed as the mean ± standard error of the mean (SEM). We tested data for normality by Shapiro-Wilk test. Independent samples *T* test was used to assess the difference between the two groups. Statistical analyses were performed using SPSS 13.0 (SPSS Inc., Chicago, IL). *P* < 0.05 (two-tailed) was considered to be statistically significant.

## Results

A total of 46 rats underwent MI surgery by ligation of the LAD coronary artery; six died after the first operation and nine rats were excluded because the results of the transthoracic echocardiography did not meet our inclusion criteria before cell grafting (eight rats: ejection fraction reduced < 15%, one rat: ejection fraction reduced > 50%). Hence, 31 rats underwent a second thoracotomy, of which 18 were placed in the iPS-CM group, involving transplantation with 1.0 × 10^7^ iPSC-CMs; 13 were placed in the control group and were injected with a 5% albumin solution. In the control group, two rats died within 24 h, and another three died within 15 days of the second operation (38.46% postoperative mortality). In the iPS-CM group, three rats were killed 24 h and 7 days after cell transplantation. In the other 12 rats, one died on the fourth day because of a cotton ball left in the chest, and another two died during the 15 days that followed the second operation (25% postoperative mortality).

### iPSC-CM injection improves cardiac function

**Fig. 1:**
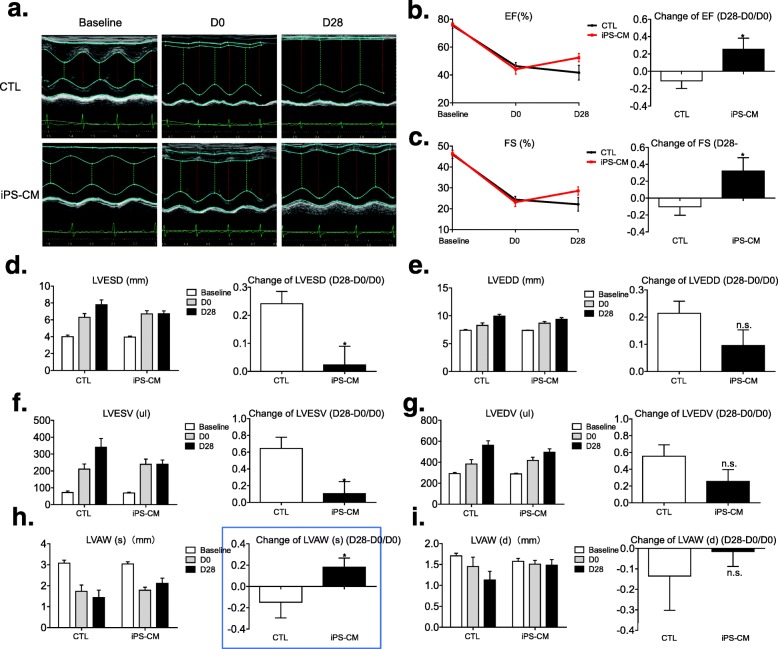
Left ventricular function following human iPSC-CM therapy. **a** Representative images of hearts at baseline (before MI model established), D0 (10 days after LAD coronary artery ligation and before iPSC-CM grafting) and D28 (4 weeks after iPSC-CM grafting) with administration of 5% albumin solution (CTL group) (upper) versus iPSC-CMs (iPS-CM group) (lower). **b** Quantification of left ventricular ejection fraction (EF) and change of EF (D28-D0/D0) demonstrated a trend toward improved functional recovery at 4 weeks after the second thoracotomy in the iPS-CM group compared with the CTL group. **c** Quantification of left ventricular fractional shortening (FS) and change of FS (D28-D0/D0) demonstrated a trend toward improved functional recovery at 4 weeks after the second thoracotomy in the iPS-CM group compared with the CTL group. **d–i** Quantification of left ventricular end-systolic diameter (LVESD), left ventricular end-diastolic diameter (LVEDD), left ventricular end-systolic volume (LVESV), left ventricular end-diastolic volume (LVEDV), end-systolic and end-diastolic left ventricular anterior wall (LVAW) thickness and changes in these indicators. iPSC-CMs induced pluripotent stem cell-derived cardiomyocytes, MI myocardial infarction, LAD left anterior descending, CTL control, LVAW(s) left ventricular anterior wall thickness (end-systolic), LVAW(d) left ventricular anterior wall thickness (end-diastolic). (CTL group: *n* = 8; iPS-CM group: *n* = 9). Unpaired two-tailed *t*-test with * vs CTL *P* < 0.05, n.s. = not significant

At 1 month after ligation, 5% albumin solution-treated infarcted rats had a significantly decreased left ventricular ejection fraction (LVEF) of 10.11% (from 46.36 to 41.67%) (Fig. 1a, b) and decreased fractional shortening (FS) of 9.23% (from 24.37 to 22.12%) (Fig. 1a, c). While animals injected with iPSC-CMs attenuated this remodeling process, improving the ejection fraction by 18.86% (from 44.09 to 52.41) (Fig. 1a, b), fractional shortening improved by 23.69% (from 23.08 to 28.54%) (Fig. 1a, c). Moreover, left ventricular end-systolic diameter (LVESD) and left ventricular end-systolic volume (LVESV) also had a significant increase in the control group and showed no significant increase in the iPS-CM group (Fig. 1a, d, f). The wall of the infarcted myocardium was significantly less thick in the control group but was improved in the iPS-CM group (Fig. 1a, h). Cell therapy tended to attenuate left ventricular end-diastolic diameter (LVEDD) and left ventricular end-diastolic volume (LVEDV) enlargement and increase the wall thickness of the infarcted myocardium in end-diastolic compared with the control group but the difference was not significant (Fig. 1a, e, g, i).

### ECG for rats during MI model establishment and 28 days after iPSC-CM therapy

**Fig. 2:**
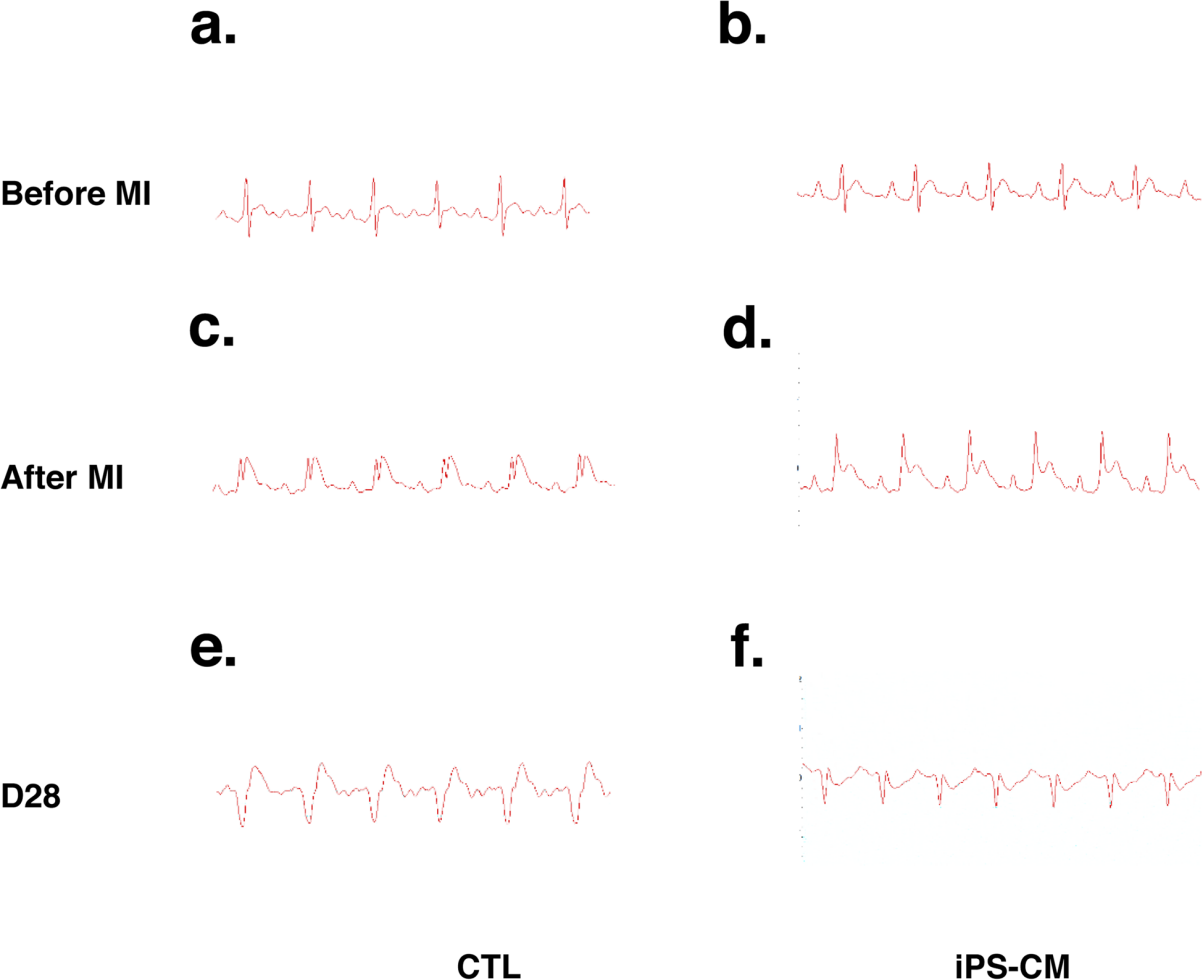
Electrocardiogram (ECG) for rats during establishment of the myocardial infarction (MI) model and 28 days after iPSC-CM therapy. Representative traces from rat ECG recordings are shown (**a**, **b**). Readings are normal at baseline before left anterior descending (LAD) coronary artery ligation in the CTL group and iPS-CM group. **c**, **d** ST segment elevation in every group after LAD coronary artery ligation, which indicated that the MI model was successful. **e**, **f** No ventricular arrhythmias occurred 28 days after iPSC-CM transplantation. CTL control, iPSC-CMs induced pluripotent stem cell-derived cardiomyocytes

ECG indicated the establishment of the MI model by ST segment elevation after LAD coronary artery ligation (Fig. 2a–d). Twenty-eight days after iPSC-CM transplantation, no ventricular arrhythmias occurred after several minutes of ECG recordings (Fig. 2e, f).

### Distribution of iPSC-CMs within rat hearts and other organs after transplantation

**Fig. 3:**
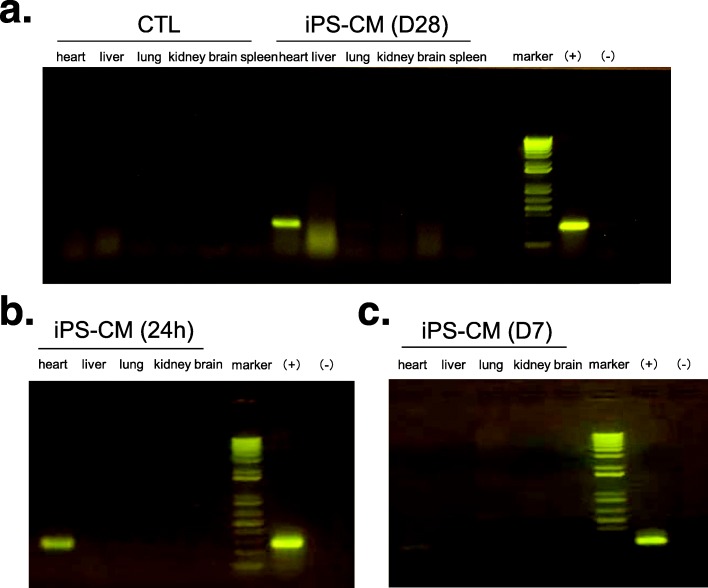
Detection of human cells within rat hearts and other organs by polymerase chain reaction (PCR) of human mitochondria DNA at different timepoints after cell transplantation. **a** PCR of human mitochondrial DNA in the heart and other organs of rats at 28 days after cell transplantation in the CTL group (*n* = 2) and iPS-CM group (*n* = 4). **b** PCR of human mitochondrial DNA in the heart and other organs of rats at 24 h after cell transplantation in the iPS-CM group (*n* = 3). **c** PCR of human mitochondrial DNA in the heart and other organs of rats at 7 days after cell transplantation in the iPS-CM group (*n* = 3). (+) indicates positive control with iPSC-CMs. (−) indicates a negative control with DEPC water. CTL control, iPSC-CMs induced pluripotent stem cell-derived cardiomyocytes

**Fig. 4:**
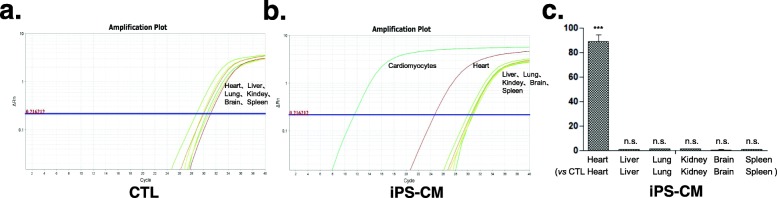
Quantitative polymerase chain reaction (qPCR) quantification of human mitochondrial DNA confirms the presence of human cells within rat hearts but not other organs 4 weeks after cell transplantation. **a** Plot of SYBR-based human mitochondrial DNA quantification demonstrated human cell distribution in each organ of rats in the CTL group. **b** Plot of SYBR-based human mitochondrial DNA quantification demonstrated human cell distribution in each organ of the iPS-CM group. Cardiomyocytes were used as a positive control. **c** qPCR quantification analysis of human mitochondria DNA demonstrated that human cells survived in rat hearts but not in other organs in the iPS-CM group (vs control group at 4 weeks following iPSC-CM transplantation) (*n* = 3 per group). CTL control, iPSC-CMs induced pluripotent stem cell-derived cardiomyocytes. Unpaired two-tailed *t*-test with * vs CTL *P* < 0.05, *** vs CTL *P* < 0.005, n.s. = not significant

PCR and qPCR-based amplification of the human mitochondria DNA within rat hearts and other organs demonstrated that most organs (liver, lung, kidney, brain, spleen) did not have signs of iPSC-CMs, but positive signals were detected in the heart at 24 h (*n* = 3), 7 days (*n* = 3), and 28 days (*n* = 4) following t0072ansplantation (Fig. 3a–c and Fig. 4a–c).

### Histological evaluation of iPSC-CMs surviving in the heart

**Fig. 5:**
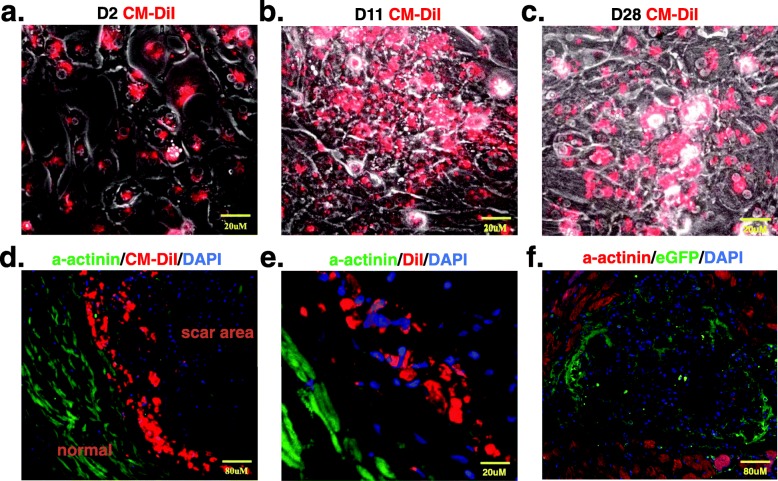
Histological evaluation confirms iPSC-CM homing to the infarcted heart. **a**–**c** The grafted cells were labeled with the fluorescent cell tracer Vybrant™ CM-DiI Cell-Labeling Solution (Molecular Probes, Invitrogen) and cultured in vitro for 2 days (**a**), 11 days (**b**), and 28 days (**c**). **d**, **e** Immunofluorescent staining shows alpha-actinin (green) in rat heart tissue; the grafted cells were prelabeled with Vybrant- CM-DiI (Red) and detected under a microscope 7 days after transplantation. **f** iPSC-CMs were detected by eGFP immunofluorescent staining 4 weeks after transplantation. iPSC-CMs induced pluripotent stem cell-derived cardiomyocytes, and eGFP enhanced green fluorescent protein

The grafted cells were labeled with the fluorescent cell tracer Vybrant™ CM-DiI, and almost all cells still had this dye after being cultured in vitro for 28 days (Fig. 5c). To track the injected cells, three rats receiving iPSC-CM injections were dissected after 24 h, and three were dissected 7 days after cell transplantation. At 1 week after cell injection, the grafted cells were detected in the rat heart by Vybrant-CM-DiI prelabeling iPSC-CMs before cell transplantation (Fig. 5d, e). Although we did not detect prelabeled Vybrant-CM-DiI cells in the rat heart 4 weeks after cell transplantation, confocal laser microscopy confirmed the presence of transplanted cells within the myocardium by eGFP immunofluorescent staining (Fig. 5f).

### Sirius red staining and hematoxylin-eosin staining

**Fig. 6:**
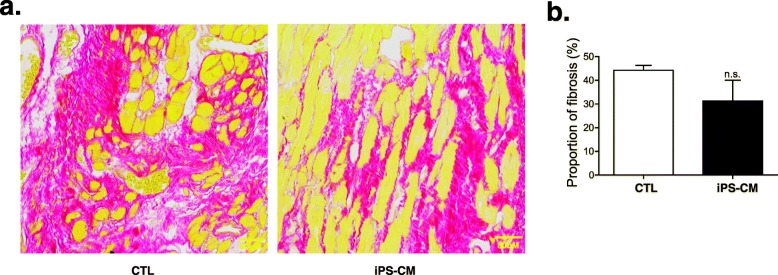
Sirius red staining to identify fibrosis. **a** Sirius red staining of cardiac sections 4 weeks after cell transplantation. Collagen fibers stain red, and cardiac muscle fibers stain yellow. **b** Quantification of fibrosis demonstrated that iPSC-CM therapy tended to reduce the amount of fibrosis compared with the CTL group, but there was no significant difference. CTL control, iPSC-CMs induced pluripotent stem cell-derived cardiomyocytes (CTL group: *n* = 5; iPS-CM group: *n* = 6). Unpaired two-tailed *t*-test, n.s. = not significant

Fibrosis was evaluated by Sirius red staining (Fig. 6a, b). There was a tendency for the iPS-CM group to have a reduced percentage of fibrosis compared with that observed in the control group, but there was no significant difference (Fig. 6b). Hematoxylin-eosin staining demonstrated no teratoma formation within the infarcted area after transplantation (data not shown).

### Tumorigenicity study

**Fig. 7:**
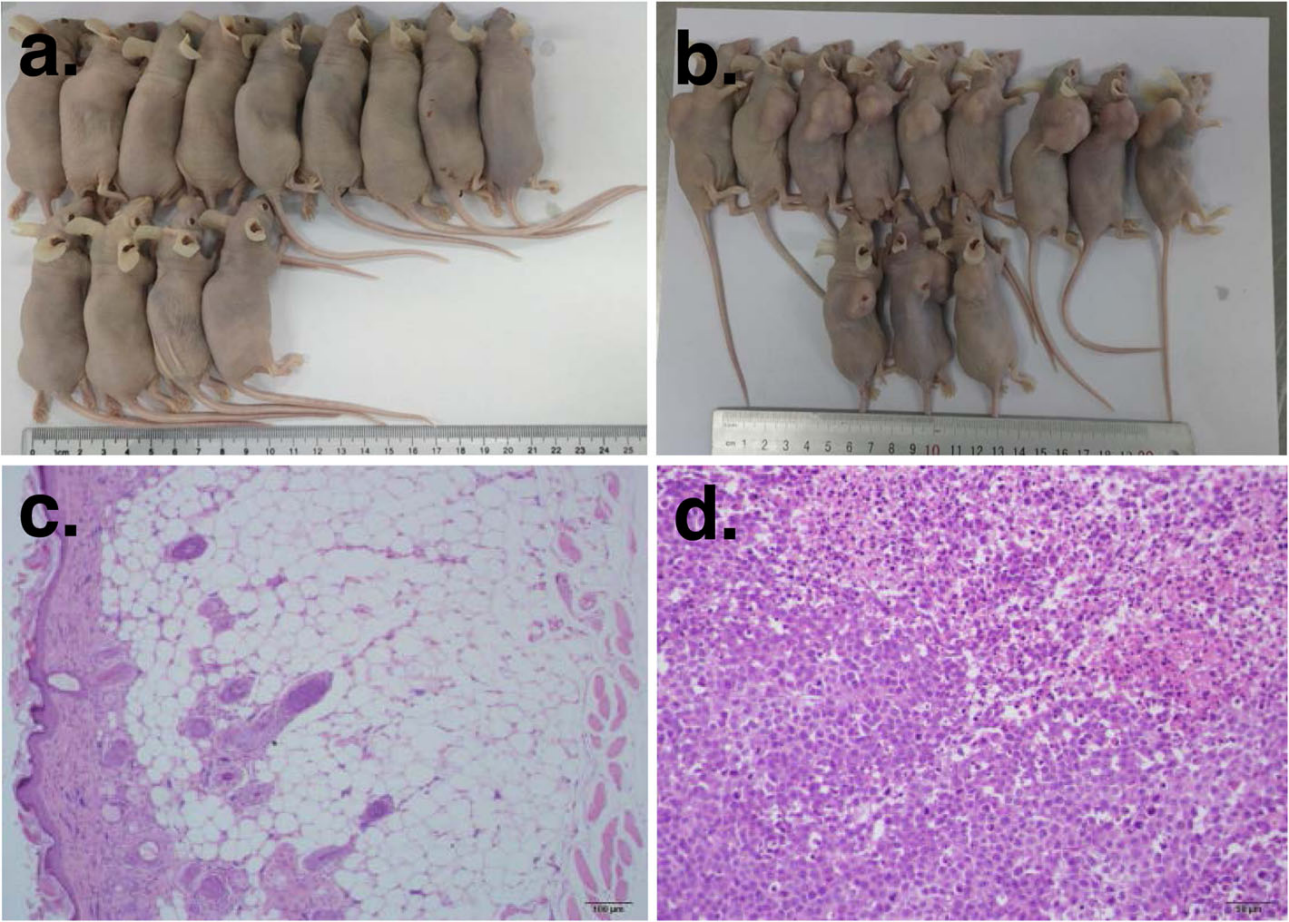
Tumorigenicity was evaluated by subcutaneous injection of 1 × 10^7^ iPSC-CMs in nude mice. **a** Thirteen Nude mice were injected with 1 × 10^7^ iPSC-CMs and did not develop tumors. **b** Twelve nude mice were injected with Hela cells (1 × 10^7^) and were used as positive control group, which developed tumor at the site of injection. **c**, **d** Hematoxylin-eosin staining of the injected site in iPS-CM group (**c**) and positive control group (**d**)

Tumorigenicity was evaluated by subcutaneous injection of 1 × 10^7^ iPSC-CMs (*n* = 13) in Nude mice. Hela cells (1 × 10^7^, *n* = 12) were used as positive control. All animals were reevaluated 4 months later. While all mice injected with Hela cells developed tumor at the site of injection (Fig. 7b, d), no rats from iPS-CM group developed tumors (Fig. 7a, c).

## Discussion

In the current study, permanent LAD coronary artery ligation was employed to establish a post-MI heart failure model in rodents. All animals developed pathophysiological changes typical of heart failure, including reduced left ventricular function and dilated cardiac geometry. Our results demonstrated that transplantation with iPSC-CMs not only significantly improved heart function in terms of LVEF and FS but also reduced overall mortality during the observation period compared with that observed in the control animals. Cardiac remodeling, including changes in ventricular volume, thickness and shape of the myocardial wall, was observed in an attempt to compensate for the reduced ventricular function [19, 22, 24]. Interestingly, implantation with iPSC-CMs rescued or even reversed the progression of cardiac remodeling. Compared with the control, the LVESD was significantly reduced after cell therapy. Similarly, left ventricular volume continued to increase throughout the observation period (Fig. 1f) in the control group. However, iPSC-CM transplantation completely stopped the worsening of left ventricular dilation, resulting in a significantly reduced LVESV. Cardiac remodeling is identified as an important marker of mortality [25, 26]. Our results demonstrated an association between higher mortality and the worsening of ventricular dilation.

Despite the promising results of PSC-CMs in heart regeneration [19, 21, 23, 24], the cellular fate of PSC-CMs after implantation has not been thoroughly assessed. Chow et al. indicated that grafted human cells were not able to be detected after iPSC-CM injection [22]. Here, we utilized three methods to monitor the engraftment of iPSC-CMs: the fluorescent cell tracer Vybrant™ CM-DiI, labeling iPSCs with eGFP expression and human mitochondrial DNA detection. The highlight of our study was providing strong evidence that grafts persisted in rat hearts after 1 month and in some individuals even 2 months (data not shown) after transplantation, but the results were based on appropriate usage of immunosuppressive agents. We found that the dose of immunosuppressants was a key factor for cell engraftment in immunocompetent animals. When immunosuppressants were reduced to half dosage, iPSC-CMs could not be detected in rat hearts 1 month after transplantation (data not shown). Four rats were sacrificed at 2 months to study long-term cell engraftment after adequate immunosuppressant treatment. However, cell grafts were only detected in one of the four rats, which may be due to rejection between different species or the position of tissue extraction. It is worth noting that the duration of Vybrant™ in vivo after 1 month remains uncertain, while human mitochondrial DNA detection by PCR is unable to confirm cell viability. In infarcted areas, immunofluorescent staining of eGFP was susceptible to nonspecific signal interference. As such, further imaging techniques, such as improved reporter gene imaging, may be required to identify the fate of stem cells in vivo [27]. Despite the strong association between cell engraftment and heart function improvement demonstrated by our results, the beneficial mechanisms may also involve paracrine effects and angiogenesis [24, 28, 29].

Cardiomyocytes derived from PSCs are a promising source of cells for heart failure therapy. Cell delivery methods were reported to be associated with cell retention [22, 30] as well as potential circulation to other organs. Hou et al. evaluated the short-term fate of peripheral blood mononuclear cells after intramyocardial, intracoronary, and interstitial retrograde coronary venous delivery in an ischemic swine model and found by whole-body γ-scans that a significant fraction of cells were delivered into the lungs [31] in each delivery method. Improving cell retention in the heart after transplantation is hypothesized to increase therapeutic efficacy. In our study, iPSC-CMs were delivered by direct intramyocardial injection during open-chest surgery, and we did not find cell retention in any organs (liver, lung, kidney, brain, spleen) except heart after 1, 7, and 28 days of cell delivery.

Ventricular arrhythmias, not surprisingly, are not detected in our study. Compared with rat myocytes, human iPSC-CMs possess much slower spontaneous beating rhythm (50–80 bpm) and prolonged action potential duration. Thus, the fast beating rat myocytes overdrives the automaticity of human iPSC-CMs, refraining the implanted cells from eliciting arrhythmogenic ectopic events.

There are several limitations in our study. First, due to technical limitations and the fast beating rate in the rodent heart failure model, MRI was not performed in the current study. However, MRI in future studies may highlight that iPSC-CM transplantation improves myocardial performance by providing infarct size and perfusion defect data. Second, the use of human iPSC-CMs to treat immunocompetent rats may induce severe rejection caused by species differences despite the use of immunosuppressants. Further study may consider either the use of infarcted RNU rats or iPSC-CMs derived from rats to reveal the long-term therapeutic effects of allograft transplantation. Third, although we demonstrated the presence of human iPSC-CMs in rat hearts by several means, future studies are needed to specifically quantify the engraftment and proliferation of cells.

## Conclusions

In summary, we report that intramuscular transplantation of human iPSC-CMs improves myocardial function, reverses ventricular remodeling, and reduces mortality in infarcted rats. We also showed that grafted cardiomyocytes can be detected in rat hearts 1 month after transplantation, which highlights the therapeutic potential of iPSC-CMs in myocardial regeneration.

## Additional file

Additional file 1 **:** Figure S1. Experimental flow chart. The myocardial infarction (MI) model was established on day − 10. Cell transplantation finished on day 0. Cardiac functional measurements were obtained at baseline, day 0 (before cell transplantation) and day 28 (days after transplantation) using echocardiography. Human cells were tracked by PCR and qPCR-based amplification of the human mitochondrial DNA within rat hearts and other organs at days 1, 7, and 28 after transplantation.

## Abbreviations

LAD: Left anterior descending
ECG: Electrocardiogram
EF: Ejection fraction
eGFP: Enhanced green fluorescent protein
ESCs: Embryonic stem cells
ESC-CMs: ESC-derived cardiomyocytes
FS: Fractional shortening
LVEDD: Left ventricular end-diastolic diameter
LVEDV: Left ventricular end-diastolic volume
LVESD: Left ventricular end-systolic diameter
LVESV: Left ventricular end-systolic volume
LV: Left ventricular
LVAW: Left ventricular anterior wall
iPSC-CMs: Induced pluripotent stem cell-derived cardiomyocytes
MI: Myocardial infarction
PCR: Polymerase chain reaction
PSCs: Pluripotent stem cells
