# Correction to: Transplantation of human induced pluripotent stem cell-derived cardiomyocytes improves myocardial function and reverses ventricular remodeling in infarcted rat hearts

**DOI:** 10.1186/s13287-020-01673-z

**Published:** 2020-05-27

**Authors:** Xumin Guan, Wanzi Xu, He Zhang, Qian Wang, Jiuyang Yu, Ruyi Zhang, Yamin Chen, Yunlong Xia, Jiaxian Wang, Dongjin Wang

**Affiliations:** 1 Department of Cardiology, The First Affiliated Hospital of Dalian Medical University, Dalian, 116011 Liaoning China; 2 Department of Thoracic and Cardiovascular Surgery, Nanjing Drum Tower Hospital, Clinical College of Traditional Chinese and Western Medicine, Nanjing University of Chinese Medicine, Nanjing, 210008 Jiangsu China; 3 Peking Union Medical College, Chinese Academy of Medical Science, Graduate School of Peking Union Medical College, Beijing, China; 4 HELP Therapeutics, Nanjing, 211166 Jiangsu China; 5 The Laboratory Animal Center, The First Affiliated Hospital of Nanjing Medical University, Nanjing, 210029 Jiangsu China; 6 Department of Cardiology, The First Affiliated Hospital of Nanjing Medical University, Nanjing, 210029 Jiangsu China; 7 Department of Cardio-Thoracic Surgery, Nanjing Drum Tower Hospital Affiliated to Medical School of Nanjing University, Nanjing, China

**Correction to: Stem Cell Res Ther (2020) 11:73**

**https://doi.org/10.1186/s13287-020-01602-0**

The original article [1] omits an affiliation for authors, He Zhang and Dongjin Wang. The omitted affiliation can be viewed in this Correction article. Furthermore, affiliation #3 has been amended accordingly.
