# Supplementary figures and images for: Transplantation of human induced pluripotent stem cell-derived cardiomyocytes improves myocardial function and reverses ventricular remodeling in infarcted rat hearts
Source: Stem Cell Res Ther. 2020 Feb 21;11:73. doi: 10.1186/s13287-020-01602-0 (PMC7033912; doi:10.1186/s13287-020-01602-0)

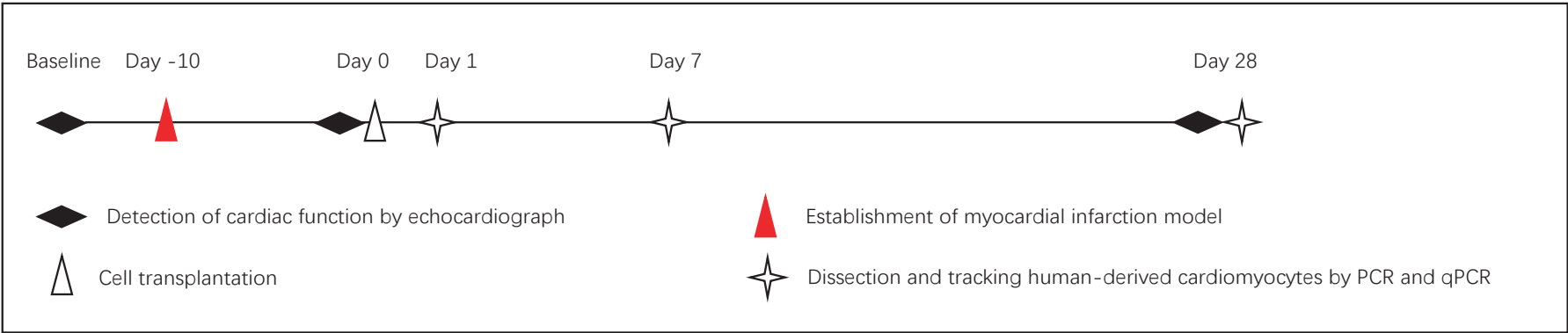

Supplement: Supplementary file 1 — : Figure S1. Experimental flow chart. The myocardial infarction (MI) model was established on day − 10. Cell transplantation finished on day 0. Cardiac functional measurements were obtained at baseline, day 0 (before cell transplantation) and day 28 (days after transplantation) using echocardiography. Human cells were tracked by PCR and qPCR-based amplification of the human mitochondrial DNA within rat hearts and other organs at days 1, 7, and 28 after transplantation. [file 13287_2020_1602_MOESM1_ESM.pdf]
